# Author's Reply

**DOI:** 10.1371/journal.pmed.0020224

**Published:** 2005-07-26

**Authors:** Shah Ebrahim

**Affiliations:** **1**University of BristolBristolUnited Kingdom

In response to Michael Makover's comments [1], the important point at issue here is *evidence*—and not whether there is room for both population approaches and high-risk approaches. We can certainly identify plaques using carotid ultrasound. We can use risk factor scoring schemes to identify those at high risk of suffering a cardiovascular event. We can give patients a range of drugs that have been shown in trials to reduce risk of these events. However, the relevant evidence from randomised controlled trials of risk factor screening (using either scores or carotid ultrasound) and intervention is simply not available. So what do we do—ignore the lack of evidence? Or do we get on with organising the trials?
